# Impact of Oral Lichen Planus on Oral Health-Related Quality of Life: A Systematic Review and Meta-Analysis

**DOI:** 10.3390/clinpract11020040

**Published:** 2021-05-07

**Authors:** Monal Yuwanati, Shailesh Gondivkar, Sachin C. Sarode, Amol Gadbail, Gargi S. Sarode, Shankargouda Patil, Shubhangi Mhaske

**Affiliations:** 1 Saveetha Dental College and Hospitals, Saveetha Institute of Medical and Technical Sciences, Saveetha University, Chennai 600077, India; monal9817@gmail.com; 2 Government Dental College & Hospital, Nagpur 44009, India; shailesh_gondivkar@yahoo.com; 3 Dr. D.Y. Patil Dental College & Hospital, Dr. D.Y. Patil Vidyapeeth, Pune 411018, India; drsachinsarode@gmail.com (S.C.S.); drgargisarode@gmail.com (G.S.S.); 4 Indira Gandhi Government Medical College & Hospital, Nagpur 440009, India; gadbail@yahoo.co.in; 5 Division of Oral Pathology, Department of Maxillofacial Surgery and Diagnostic Sciences, College of Dentistry, Jazan University, Jazan 45142, Saudi Arabia; 6 People’s College of Dental Sciences & Research Centre, Bhopal 462037, India; shubhashok2@gmail.com

**Keywords:** oral health-related quality of life, oral lichen planus, OHIP-14, systematic review and meta-analysis

## Abstract

Oral health-related quality of life (OR-QoL) measurement in patients with oral lichen planus (OLP) can provide valuable information for the optimal management of their clinical conditions. The main objective of the present study was to assess the OR-QoL of patients with OLP as measured by the short-form Oral Health Impact profile-14 (OHIP-14) questionnaire. PubMed/MEDLINE, ISI/Web of Science, clinical trial registry, Embase, Scopus, and grey literature (via Google Scholar and Scilit) were searched. Reviewers independently screened titles/abstracts, assessed full-text articles, extracted data, and appraised their quality. Random effect analysis along with subgroup analysis for age, gender, and clinical type was performed. Seventeen studies were included. Mean overall OH-QoL was 15.20, [95% CI 12.176, 18.231]; a higher OHIP-14 score was seen in OLP patients, resulting in poor OH-QoL. The impact of OLP on OH-QoL life was moderate as compared to healthy subjects. However, medical treatment of the disease improved the OH-QoL and thus reduced the impact of OLP on it. OH-QoL among patients with OLP is generally poor. Clinicians and physicians should consider the OH-QoL of these patients as part of patients’ evaluation and modulate the administered treatment based on the OH-QoL response.

## 1. Introduction

Oral lichen planus (OLP) is a chronic immunological disease affecting oral health-related quality of life (OH-QoL). OLP prevalence is low (1.01%); however, it is of great concern to patients as it affects their quality of life (QoL) [1]. OLP is clinically characterized by pain, burning sensation, and discomfort to the individuals suffering from it [2]. These clinical symptoms may make patients prone to the development of stress, anxiety, and depression [3]. OLP is treated with a variety of medicines and agents to achieve systematic relief or complete resolution [4]. Unfortunately, these interventions mostly provide the objective evidence of benefits in the majority of clinical studies and can give a misleading sense of improvement in OLP and QoL. Besides, researchers considered the different outcomes while evaluating OH-QoL in OLP in various studies [5]. Furthermore, these studies had no uniform definition for those outcomes and used different QoL tools for measurements. Hence, it is difficult to make comparisons between these studies. One of the core issues in these studies was the non-uniform utilization of the QoL assessment instrument. This brings up the important dilemma of selecting Patient Reported Outcome Measures (PROMs) for assessment of OH-QoL in OLP patients. At present, there is no definitive choice of PROM. Another factor that causes this dilemma is the lack of focus by researchers on OH-QoL in OLP clinical studies and the lack of enough evidence for any single QoL tool [5]. Clearly, there is a need of defined outcome measures and a valid QoL measurement instrument.

The Chronic Oral Mucosal Diseases Questionnaire (COMDQ), Oral Health Impact Profile (OHIP)-49, and OHIP-14 are commonly used PROMs [6]. Each have their merits and demerits, there is no consensus on preference for any of them; however, OHIP-14 is the most commonly employed QoL PROM in clinical trials [6]. Easy use, reliability, validity, and availability of OHIP-14 in different languages make it more practical in simple settings. Another instrument, COMDQ-15, a short version of the original COMDQ, is suggested for evaluating the QoL in OLP patients; however, it is less utilized in clinical studies. Further, it requires additional testing with regard to psychosomatic properties and interpretation [6]. These commonly used PROMS are non-comparable because of heterogeneity in outcome measures and measurement methods. These issues make it difficult for clinicians to select an appropriate QoL or PROM tool to measure the impact of interventions on patients’ OH-QoL. Hence, we decided to focus on studies evaluating OH-QoL using the OHIP-14 tool for the present review, to address the above-mentioned issues and provide evidence on OH-QoL in OLP assessed using OHIP-14.

### 1.1. Research Question

We formulated primary research question(s) according to PICOS: 1. What is the impact of oral lichen planus on oral health-related quality of life assessed by OHIP-14? We also planned to address secondary questions, if adequate data were available for analysis: 1. What is the mean score for OHIP-14 in OLP patients? 2. Does treatment improve OH-QoL? 3. Which independent variable (clinical type, gender, age) has greater correlation with OH-QoL in OLP patients?

### 1.2. PICOS

Participants: patients with OLP, regardless of gender, age, race, clinical subtype(s), or disease severity.

Intervention: any standard treatment, without/no treatment, symptomatic treatment.

Comparator: healthy individuals or any control group, without control group, before and after.

Outcome: Oral Health-Related Quality of life and OHIP-14 as main outcome or one of the reported outcome.

Study Setting: observational, cross-sectional, case–control, and cohort studies and randomized and non-randomized clinical trials.

## 2. Materials and Methods

A systematic review and meta-analysis of OH-QoL in OLP were carried out according to established Preferred Reporting Items for Systematic Reviews and Meta-Analyses (PRISMA) guidelines (Table S1) [7,8].

### 2.1. Systematic Literature Searches and Eligibility Criteria

The target population was patients with OLP enrolled from primary care, hospital, or general population. We included studies that assessed OH-QoL in OLP in adult patients regardless of gender, age, race, clinical subtype(s) or disease severity, type of treatment (standard treatment, without/no treatment, symptomatic treatment). We included studies that were conducted without a control group or with comparisons with a control group or between treatments or with pre-treatment post-treatment groups. Only fair and good-quality observational, cross-sectional, case-control, cohort studies, randomized/non-randomized clinical trials, and case series with a minimum of 10 cases were included. Studies that reported OHIP or OH-QoL as main outcome or one of the outcomes in OLP patients were also considered for inclusion.

To address the research question(s), we specifically sought studies on OH-QoL in OLP. Two authors systematically searched PubMed, Scopus, ISI/Web of Science, EMBASE, Clinical trial registry, and grey literature (via Google Scholar and Scilit) for OH-QoL to retrieve relevant articles. Search strategies included a combination of keywords, MeSH (Medical Subject Headings) and glossary terms relevant to any published studies of OLP evaluating OH-QoL. The searched publications were only considered if written in English language, with no restrictions on year of publication. The following search strategy was constructed: (Oral lichen planus OR “Oral lichen planus” OR lichen planus OR OLP) AND (quality of life OR QoL OR “QoL” OR “oral health-related quality of life” OR OHRQoL OR OHIP-14 OR Oral Health Impact Profile) (Table S2). Reference lists of all selected articles were screened manually to identify additional studies left out in the initial search. Mendeley Desktop (Version 1.19.6) reference manager software was used to manage references.

Studies were included if they met the following criteria: (1) observational, cross-sectional, case–control, cohort studies, randomized/non-randomized clinical trials, and case series with a minimum of 10 cases; (2) OH-QoL in OLP patients assessed using the OHIP-14 as the main outcome or one of the outcomes; (3) a sample size of 10 or more patients and (4) OHIP-14 recorded as mean score with standard deviations (SDs) or standard errors (SE).

Studies were excluded if: (1) were case series with less than 10 cases, case reports, letters to editor, and correspondence; (2) were duplicates using the same patient data; (3) did not assessed OH-QoL using OHIP-14; (4) neither recorded OHIP-14 summary score or subdomain score, with means, SDs, or SE.

### 2.2. Data Extraction

Two authors independently extracted data from each included study: first author, publication year, sample size, gender and mean age of the participants, clinical information, treatment or interventions, sample size, mean overall OHIP-14 score (overall, subdomain score), follow-up period. Any disagreement was resolved by discussion until consensus was reached or by consulting a third author.

### 2.3. Quality Assessment

Two authors independently assessed the risk of bias of each study using a relevant risk of bias (RoB) tool based on study design (MINOR tool for non-randomized trials or Revised Cochrane risk of bias tool for randomized trials (RoB 2.0) or the NIH Quality Assessment Tool for Observational Cohort and Cross-Sectional Studies. Disagreements were resolved by discussing with a third author.

### 2.4. Strategy for Data Synthesis

The statistical analyses were performed using Review Manager (RevMan) [Computer program] Version 5.4 (Academic Use), The Cochrane Collaboration, 2020 and OpenMeta [Analyst] [9]. The included studies were analyzed both qualitatively and quantitatively. A narrative synthesis of the findings was provided concerning the OH-QoL outcomes in OLP. We calculated the standardized mean differences (SMD) with 95% CI for continuous data (OHIP score). In primary studies were overall mean OHIP was not provided, it was extrapolated using the subdomain score. Heterogeneity of studies was assessed by the chi-square test (P < 0.1 indicating statistical significance) and I² statistic (a quantitative measure of inconsistency among studies).

The random-effect model was conducted to pool data regardless of heterogeneity. Funnel plot was used to assess the publication bias. Additionally, subgroup analysis was conducted for gender, age clinical types of OPL, intervention, if adequate data were available. To assess heterogeneity, meta-regression analyses were performed based on age, female proportion, sample size, and publication year.

## 3. Results

### 3.1. Search Results

**Figure 1:**
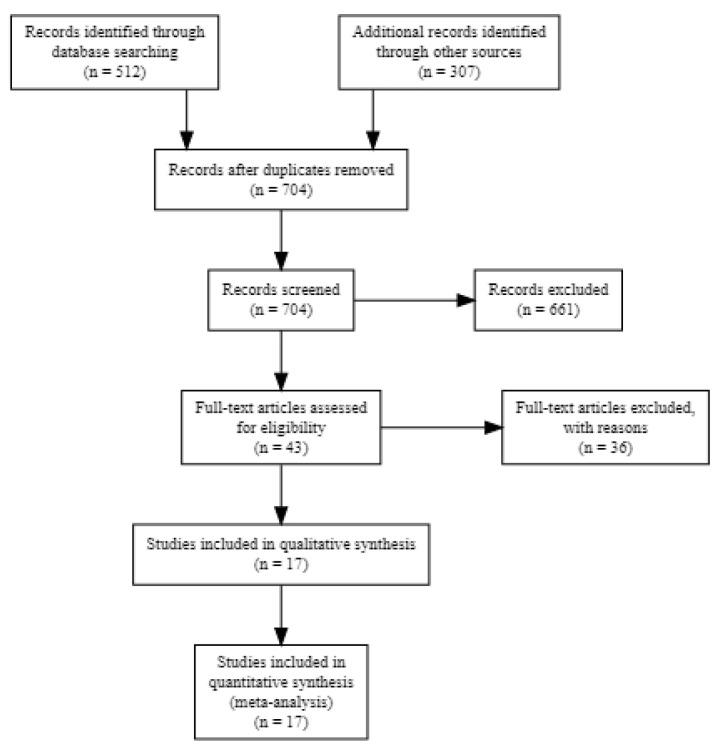
Study selection procedure (PRISMA flow Chart).

We screened 704 potentially relevant, non-duplicate articles (Figure 1). In total, 661 articles were excluded based on an initial screening of study titles and abstracts. Additional screening after obtaining the full texts of the articles was carried out for eligibility, which resulted in the exclusion of additional 31 articles due to them not meeting the eligibility criteria for inclusion. After data collection, additional 5 studies were excluded, resulting in 17 articles [10,11,12,13,14,15,16,17,18,19,20,21,22,23,24,25,26] being deemed eligible for inclusion. Among the included studies, 14 articles reported the overall OH-QoL score as a mean and standard deviation. The OHIP-14 score for two studies were obtained from the authors, whereas it was extrapolated from median/range values for four studies.

Total participants with OLP were 1265 (pooled mean sample size 54.9 ± 62.1; median 44, range 10–300), with pooled mean age of 56 years (pooled SD; 6.3 years), and the percentages of males and females in the sample were 35.73% (452) and 64.27% (813), respectively. OH-QoL was evaluated either as primary or secondary outcome along with other PROMs such as Visual Analogue Scale for pain, McGill pain score, Severity Score, HAD (anxiety and depression), GHQ-12, SF-36 (Table 1). Six studies evaluated the effect of drug treatment on OH-QoL in OLP patients. Four studies compared either baseline or end-of-treatment/follow-up OH-QoL with those of control/healthy subjects. (Table 2 and Table 3).

**Figure 2:**
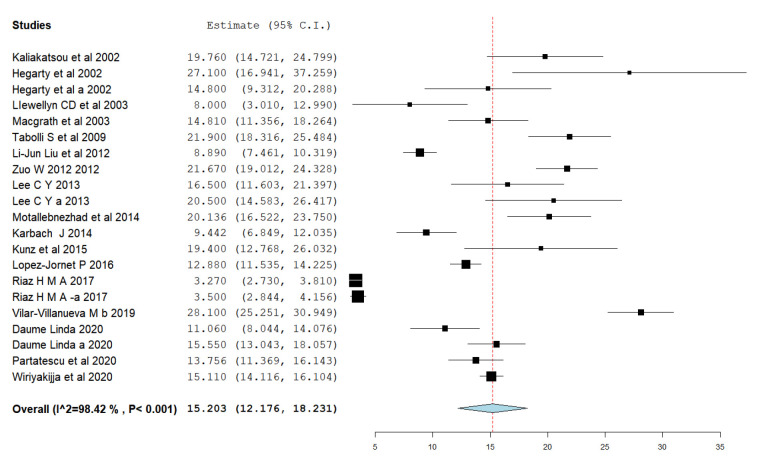
Baseline OHIP-14 score in OLP patients.

The pooled effect estimate (Baseline) (Table 4) calculated using the random-effects method (DerSimonian and Laird method) was computed to be 15.20, [95% CI 12.176 and 18.231], with a statistically significant heterogeneity (I^2^ = 98.42%; *p* < 0.0001) (Figure 2).

**Figure 3:**
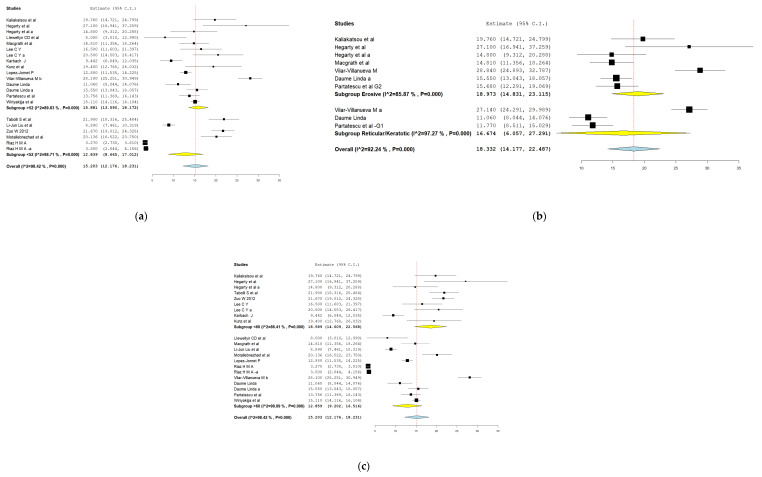
(**a**): Subgroup analysis based on age. (**b**): Subgroup analysis based on clinical type. (**c**): Subgroup analysis based on female proportion in the sample.

**Figure 4:**
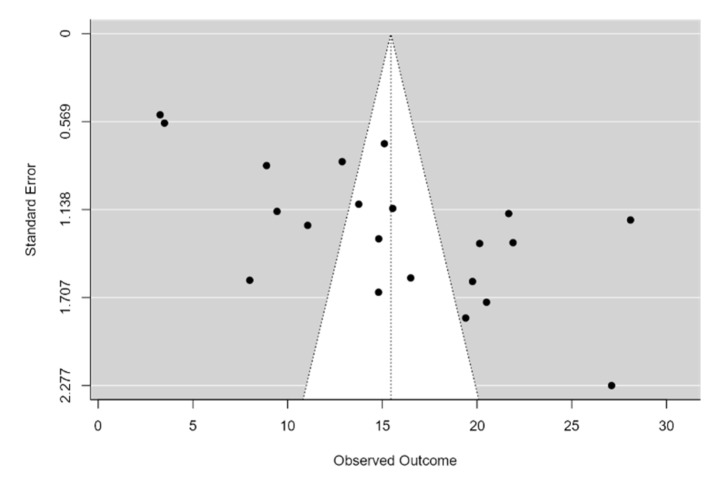
Funnel plot for publication bias.

Considering the high degree of heterogeneity, subgroup analysis based on age (>52 vs. <52 years), female proportion (>60% vs. <60%), and clinical type (keratotic/reticular vs erosive/ulcerative/bullous) was conducted (Figure 3a–c). The test for age subgroup differences suggested no statistically significant subgroup effect (*p* = 0.21), meaning that age does not modifies the impact of OLP on OH-QoL. Subgroup analysis for female proportion in samples showed a statistically significant subgroup effect (*p* = 0.07), meaning that gender statistically significantly modifies the impact of OLP on OH-QoL. The impact of OLP on OH-QoL appeared greater in studies with a larger number of females than males; therefore, the subgroup effect is quantitative. Few studies analyzed OH-QoL in the different clinical forms of OLP. Subgroup analysis based on clinical forms showed no statistically significant effect of clinical type of OLP on OH-QoL (*p* = 0.84). There was no difference in OH-QoL between reticular, keratotic, erosive, bullous, atrophic types of OLP. Egger’s test for a regression intercept was significant (*p* < 0.001), indicating evidence of publication bias (Figure 4).

**Figure 5:**
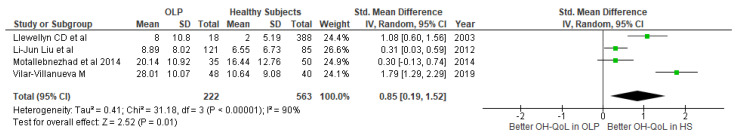
OHIP Score in OLP patients vs. Healthy subjects.

A meta-analysis of OH-QoL in OLP patients versus healthy patients showed poor OH-QoL (SMD 0.85, 95% CI, 0.19 1.52, *p* = 0.01, I^2^ = 90%) in OLP patient as compared to healthy controls (Figure 5). This confirmed our finding that OLP has an impact on OH-QoL. When the effect of treatment on OH-QoL in OLP was evaluated, it was observed that the treatment could improve the OH-QoL. However, this was applicable only to the symptomatic type of OLP, considering that most studies included the symptomatic type of OLP in their analyses. Further, a comparison between asymptomatic and symptomatic OLP should be performed.

A meta-regression was carried out for publication year, sample size, mean age, and female proportion. Findings of the meta-regression analysis stratified according to the year of publication and to the sample size are shown in Figures S1–S4. Both meta-regressions were not statistically significant (*p* value: publication year = 0.685; sample size = 0.873; age = 0.740; female proportion = 0.832).

### 3.2. Quality Assessment

A quality assessment of the 17 articles was carried out (Tables S3–S5). According to the RoB-I tool, out of four RCT studies included in this review, two studies showed high risk of bias, whereas other two showed some concerns. Four non-randomized studies were assessed using MINOR criteria; three were of low quality. Ten observational studies were assessed using the NIH Quality Assessment Tool for Observational Cohort and Cross-Sectional Studies. Nine studies were rated good.

## 4. Discussion

OH-QoL has become an important aspect in oral diseases management, considering the impact of these disease on general health and well-being [27]. OLP causes pain and discomfort to patients, resulting in poor OH-QoL; clinicians use various treatment modalities to treat it; however, most of the times, it is difficult to evaluate the effect of treatment on OH-QoL due to variations in objective, baseline reference points, and QoL tools. This systematic review and meta-analysis aimed to evaluate the impact of OLP on OH-QoL assessed by OHIP-14.

Our results showed that OLP has a negative impact on OH-QoL. Further, OLP patients have poor OH-QoL compared to healthy subjects. This is mainly attributed to pain and a burning sensation, further complicated by difficulties faced by physicians in providing a satisfactory treatment as a result of the unclear etiopathogenesis, variable clinical sign, and symptoms of this disease [28,29]. Hegarty et al. evaluated for the first time the QoL of OLP patients using OHIP-14 and OHQoL index [10]. According to them, the occurrence of OLP causes oral health consequences, which influence OH-QoL, and hence, suggested the development and use of OLP-specific PROMs [30]. OHIP-14, OHIP-49, COMDQ-15, SF-36, OHQoL-Uk-16 were used to assess the QoL in OLP patients [14]. Of these PROMs, OHIP-14 and OHIP-49 re the most commonly used PROM. OHIP-14 is the preferred one among clinicians and patients, besides being shorter and reliable. Nevertheless, some clinicians considered it to be non-specific for OLP and thus do not apply it with OLP patients. Even with these disadvantages, OHIP-14 can record the OH-QoL adequately with reliability [10,12] and shows a strong agreement with the disease-specific COMDQ-15 questionnaire [31]. Measuring the OH-QoL at baseline can help clinicians plan patient-oriented treatments.

Baseline OH-QoL was variable in the included studies, which could be due to heterogeneity of the OLP population, different diagnostic criteria, and different clinical forms of OLP. Moreover, several factors can influence the OH-QoL. Symptomatic OLP has a greater negative impact on OH-QoL as compared to asymptomatic OLP [17]. Erosive/ulcerative, bullous, and atrophic OLP cause a burning sensation and pain; thus, these patients have a poor OH-QoL. Vilar-Villanueva et al. found a higher OHIP-14 score for atrophic/ulcerative OLP patients as compared to patients with reticular OLP [21]. Karbach et al. reported similar findings (symptomatic vs. asymptomatic OLP) with lower OHIP-14 score [17]; however, Parlatescu et al. did not find a significant difference between asymptomatic and symptomatic OLP patients [23]. They attributed this observation to the low number of clinical subtypes of OLP in their study, but Wiriyakijjia et al. study, based on a large number of clinical subtypes of OLP, observed higher QoL score/poor QoL in erosive/ulcerative OLP patients than in keratotic OLP patients [32]. Hence, a sufficient number of clinical subtypes should be included to get precise estimation of OH-QoL.

OH-QoL in OLP patients differs depending on gender. The female gender generally presents a poor OH-QoL [17]. Only Karbach et al. and Wiriyakijjia et al. compared the OH-QoL between males and females [17,32]. Karbach et al. reported poor Oh-QoL in females with OLP [17], whereas Wiriyakijjia et al. and Saimadhavi et al. observed a non-significant difference in the OH-QoL between males and females [32,33]. This difference could be due to the different proportions of females in these studies, which could influence the overall OH-QoL reported. Further, females are more likely to seek clinicians, especially dentists, for early intervention [34,35,36,37]. This could partially justify the higher number of females in the sample. Considering the difference in OH-QoL between males and females, clinicians should modulate the treatment plans to improve QoL in OLP patients. Further, the treatment of female patients may require a different approach, considering the increase risk of malignant transformation [34].

Researchers have used different study designs to assess the OH-QoL in OLP patients (Table 1). The most common ones are cross-sectional, observational, and single-group study design with a single outcome related to OH-QoL. These studies involved only OLP patients without any control group or comparator and measured OH-QoL at the time of diagnosis or prior to the onset of treatment; only few studies also involved control or healthy subjects. Although randomized clinical trial for OLP have been conducted, OH-QoL was measured as a secondary outcome [16,30]. Further, these studies mostly concentrated on evaluating the efficacy of drugs/medicines for symptomatic relief in patients; few authors evaluated the clinical improvement in signs or lesions [38].

Over the decades, clinicians have been treating OLP using various modalities, mostly using drugs [4]. Unfortunately, there is no definitive curative treatment for OLP at present, and the available treatment modalities are not effective in achieving complete resolution of OLP. Instead, OLP can undergo remission, recurrence, or exacerbation, leading to poor OH-QoL. Clinicians have used topical ointments, sprays, and injections to treat OLP or systemic drug therapies, albeit limited success [4]. In addition, they have observed improvement in OH-QoL. The present meta-analysis reports similar observation, indicating that OH-QoL in OLP patients showed improvement irrespective of type of drug treatment. Furthermore, PROMs were found sensitive to treatment effect [12]. However, this needs to be further researched, because the presence of symptoms can influence the response to PROMs.

OHIP-14 is a reliable and valid PROM used in the past to measure the impact of OH-QoL in various oral diseases and conditions [39,40,41]. Even though it was not developed specifically for the assessment for oral mucosal diseases such as OLP, it has proven its reliability and validity in assessing the OH-QoL in OLP patients [10]. The OHIP-14 score in OLP patient has not been standardized yet; however, it is lower as compared to that of normal individuals. The pooled mean OHIP-14 score in OLP patients in our meta-analysis was higher than that in the normal population. However, it is difficult for clinicians and patients to notice a definitive change or improvement in OH-QoL after interventions. Wiriyakijjia et al. suggested a threshold for minimal important changes in QoL score [25]. The OHIP-14 scores for OLP patients in the included studies were variable. After a medicinal intervention, there was an improvement in the OH-QoL, with a medium effect as compared to baseline. However, the number of days or weeks after which a change or improvement in OH-QoL was noticed was not indicated. Robust randomized clinical drug trials are required to know more about changes in OH-QoL, with a standardized follow-up.

The present meta-analysis has few limitations. We observed high heterogeneity within the included studies. The reasons for it could be different study designs, recruitment processes, target populations. The studies were conducted on hospital patients or visiting patients (outpatient) or on patients recruited randomly in clinical trials [14,15,19]. Most of these studies included more female participants, which limited the analysis on relation to gender. Future studies with equal gender representation should be conducted. The variability in clinical characterization as well as diagnosis of OLP in primary studies could have influenced the results of this meta-analysis. Selective inclusion of symptomatic OLP patients, socioeconomic status, occupation, and mental status could be responsible for the observed heterogeneity [21].

## 5. Conclusions

OLP has considerable impact on OH-QoL, irrespective of age and gender. In spite of fewer treatment modalities, medical interventions improve the OH-QoL of OLP patients. Clinicians should incorporate a OH-QoL assessment using PROMs such as OHIP-14 when establishing a treatment protocol for OLP patients, to monitor treatment outcomes. OH-QoL assessment using OHIP-14 can assist in monitoring and modulating an administered treatment. Considering the chronic nature of OLP, longitudinal cohort studies with long-term follow-up should be conducted to observe the overall trend in OH-QoL in OLP patients.

## Figures and Tables

**Table 1:** Characteristics of the included studies that assessed Oral Health-Quality of Life associated with oral lichen planus using the Short-Form Oral Health Impact Profile (OHIP)-14.

Year	Author	Symptomatic/Asymptomatic	Clinical Type		PROM	Study Design			Language	Country
2002	Hegarty et al.	Symptomatic	Erosive (36); Ulcerative (12)		OHIP-14, OHQOL-UK, VAS	Prospective study; Single group			English	UK
2002	Kaliakatsou et al.	Symptomatic	Erosive; Ulcerative		OHIP-14; VAS-Pain; McGill pain score	Prospective study; Single group; Before and After			English	UK
2003	Macgrath et al.	Symptomatic	Erosive (34); Ulcerative (10)		OHIP-14; VAS-Pain; OHQoL-Uk-16	Prospective study; Single group; Before and After			English	UK
2003	Llewellyn, S. et al.	Not mentioned	Not mentioned		OHIP-14; HAD; VAS	Cross-sectional, Single group			English	UK
2009	Tabolli, S. et al.	Not mentioned	Not mentioned		OHIP-14, SF-36, GHQ-12	Prospective, Observational, Single arm study			English	Italy
2012	Li-Jun Liu et al.	Not Mentioned	Not Mentioned		OHIP-14 (Chinese Version), SF-36 (Chinese Version)	Observational, Two-Arm			English	China
2013	Lee, Y.C. et al.	Not Mentioned	Not mentioned		OHIP-14	Prospective RCT			English	Korea
2014	Karbach, J. et al.	Not mentioned	Not mentioned		OHIP-14	Prospective study			English	Germany
2015	Kunz et al.	Not mentioned	Not mentioned		OHIP-14	Prospective, Open label (Pilot)			English	Switzerland
2016	Lopez-Jornet, P. et al.	Not mentioned	Not mentioned		OHIP-14	Randomized Double blind- Parallel group			English	Spain
2017	Riaz, H.M.A. et al.	Not mentioned	Erosive/Ulcerative (P = 11; T = 13) Erythematous/Atrophic (P = 3; T = 1) Reticular (P = 5; T = 6)		OHIP-14	Randomized Clinical trial (Pimecrolimus (P) vs. Triamcinolone (T)			English	Pakistan
2019	Vilar-Villanueva, M.	Not mentioned	Atrophic/Ulcerative (25) Reticular (23)		OHIP-14, HADS	Cross-sectional, Observational			English	Spain
2020	Daume, L. et al.	Not mentioned	Keratotic (50) Erosive (62)		OHIP-14, VAS	Prospective Observational			English	Germany
2020	Parlatescu et al.	Not mentioned	Keratotic (39) Atrophic (14) Bullous (02)		OHIP-14, VAS	Cross-sectional, Single centre			English	Romania
2012	Zuo, W. et al.	Not mentioned	Not mentioned		OHIP-14	NA			Chinese	China
2020	Wiriyakijja et al.	Not mentioned	Keratotic (51) Erythematous (201) Erosive/ulcerative (44)		OHIP-14	Cross-sectional			English	Ireland
2014	Motallebnezhad et al.	Not mentioned	Not mentioned		OHIP-14	Observational			English	Iran
** Year **	** Author **	** Age (Mean ± SD (Range)); Years **	** Gender ** ** (Female) **	** OLP cases (Initially ** **Recruited/Sample Size)**	** Intervention/Drug** **Treatment**	** Comparator/Control **	** Aspects ** ** Studied **	** OHIP-14 ** ** Assessment ** ** (Follow-up) **	** Other/Comparator PROM **	** Remark **
2002	Hegarty et al.	Mean 54.15 ± 12.85) MD 53 (26–86)	10 (38)	48 (48)		None	OH-QoL, Pain	Baseline	OHQoL-UK VAS	
2002	Kaliakatsou et al.	62 (28–87)	5 (14)	19 (19)	0.1% topical Tacrolimus	Follow-up/End of Treatment	OH-QoL, Pain	Baseline; Follow-up (1, 3, 5, 7, 8 & 10, 14, 18, 22 weeks)	VAS; McGill Pain Score	Data extrapolated
2003	Macgrath et al.	54.55 ± 12.70 MD (53 (46.3–60.0)	9 (35)	44 (48)	0.5 mg Tab Betamethasone	End of treatment	OH-QoL, Pain	Baseline; End of treatment	VAS; McGill Pain Score; OHQoL-UK-16	
2003	Llewellyn, S. et al.	60.3 ± 17.4	4 (14)	18 (140)		None	OH-QoL, Pain, Anxiety, Depression	Baseline	VAS; HAS (Anxiety and Depression)	Data extrapolated
2009	Tabolli, S. et al.	Not mentioned	Not mentioned	49 (206)		None	OH-QoL, HR-QoL,	Baseline	SF-36, GHQ-12	
2012	Li-Jun Liu et al.	OLP = 49.32 ± 16.18 (19–83) HS = 46.34 ± 16.69	OLP = 33 (88); HS = 31 (54)	121 (121)		Health Subjects	OH-QoL, Generic QoL	Baseline	SF-36	
2013	Lee, Y.C. et al.	56.6 ± 11.7 (MR); 57.1 ± 6.6 (IL)	11 (7) (MR); 9 (11) (IL)	38 (40)	Triamcinolone acetonide	Follow-up/End of treatment	Pain, OH-QoL,	Baseline, Follow-up	VAS	
2014	Karbach, J. et al.	64 (13.81; 35 to 88)	22 (15)	37 (154)		None	OH-QoL	Before/ Prior Treatment	-	
2015	Kunz et al.	55.6 ± 16.6	6 (4)	10 (10)	Oral Alitretinoin	Follow-up/End of Treatment	OH-QoL, Severity Score	Baseline; Follow-up (12, 24 wks)	ESS	
2016	Lopez-Jornet, P. et al.	63.1 ± 14.3 (Rx) 62.8 ± 10.3 (P)	10 (16) (Rx); 7 (22) (Plc)	55 (70)	Topical 2% chamaemelium nobile	Follow-up/End of Treatment	Pain, OH-QoL, Anxiety, Depression	Follow-up (4 wks)	VAS, HASD	
2017	Riaz H M A et al.	44.50 ± 6.20 (P) 45.72 ± 5.35 (T)	2 (16) (P); 6 (12) (T)	36	Pimecrolimus vs. Triamcinolone	Follow-up/End of Treatment	Pain, OH-QoL	Follow-up (4 months)	VAS-Pain	
2019	Vilar-Villanueva, M.	59.7 (OLP); 61 (C)	7 (41) (OLP); 15 (25) (HS)	88 (88)	None	Health Subjects	Pain, OH-QoL	Baseline	HADS	Data obtained from author
2020	Daume, L. et al.	59.98 ± 10.69	21 (91)	112 (112)	None	None	Pain OH-QoL	Baseline	VAS	
2020	Parlatescu et al.	64.14 ± 11.63 (OLP) 55.13 ± 12.64 (HS)	16 (64) (OLP) 80 (HS)	160 (160)	None	Healthy subjects	Pain OH-QoL	Baseline	VAS	Data extrapolated (pooled mean & SD)
2012	Zuo, W. et al.	NA	24 (27) (OLP)	51(51)	None	None	Pain OH-QoL	Baseline	VAS	Data extracted from Abstract
2020	Wiriyakijja et al.	63.2 ± 11.5 (22–88)	66 (234)	300 (300)	None	NA	OH-QoL	Baseline	-	Data extrapolated (pooled mean & SD)
2014	Motallebnezhad et al.	42.22 ± 9.97 (C) 44.17 ± 14.07 (OLP)	6 (29) (OLP) 9 (41) (C)	35	None	Control	OH-QoL	Baseline	-	Data obtained from author

ESS, Escudier Severity Score; OHIP; VAS; HADS; SF; HS, Healthy Subject; P, Placebo; OLP, Oral Lichen Planus; C, Control; Rx, Treatment group.

**Table 2:** Studies that compared Oral Health-Quality of Life (baseline) between patients with oral lichen planus and healthy controls using the Short-Form Oral Health Impact Profile (OHIP)-14.

Name of Study	Year	Oral Lichen Planus		Health Control	
		Mean ± SD	n	Mean ± SD	n
Li-Jun Liu et al.	2012	8.89 ± 8.02	121	6.55 ± 6.73	85
Vilar-Villanueva, M.	2019	28.01 ± 10.07	48	10.64 ± 9.08	40
Karbach et al.	2014	9.42 ± 11.4	73	6.30 ± 7.46	12,932
Llewellyn, C.D. et al.	2003	8.00 ± 10.8	18	2.00 ± 5.19	388
Motallebnezhad et al., 2014	2014	20.14 ± 10.92	35	16.44 ± 12.76	50

**Table 3:** Studies that compared Oral Health-Quality of Life (Pre-Post) in oral lichen planus patients before and after intervention plans using the Short-Form Oral Health Impact Profile (OHIP)-14.

Name of Study	Year	Pre-Treatment		Post-Treatment/Follow-up	
		Mean	n	Mean	n
Kaliakatsou et al., 2002	2002	19.76 ± 10.6	17	8.94 ± 10.63	17
Macgrath et al., 2003	2003	14.81 ± 12.21	48	11.27 ± 10.2	48
Kunz et al., 2015	2015	19.4 ± 10.7	10	11.4 ± 8.9	10
Lopez-Jornet, P. 2016	2016	12.88 ± 3.5	26	8.38 ± 3	26
Riaz, H.M.A. 2017	2017	3.27 ± 1.17	18	1.45 ± 1.03	18
Riaz, H.M.A. -a 2017	2017	3.5 ± 1.42	18	1.45 ± 1.08	18

**Table 4:** Oral Health-Quality of Life associated with oral lichen planus using the Short-Form Oral Health Impact Profile (OHIP)-14 (Baseline).

Name of Study	Year	Mean ± SD	Sample	Mean [95% CI]	Remarks
Hegarty et al.	2002	27.1 ± 31.1	36	27.1 [16.941, 37.259]	Single study different group but same condition
Hegarty et al.	2002	14.8 ± 9.7	12	14.8 [9.312, 20.288].	
Kaliakatsou et al.	2002	19.76 ± 10.6	17	19.76 [14.7212, 24.7988].	
Macgrath et al.	2003	14.81 ± 12.21	48	14.81 [11.3558, 18.2642].	
LIewellyn, C.D. et al.	2003	8.00 ± 10.8	18	8 [3.01, 12.99]	
Tabolli, S. et al.	2009	21.9 ± 12.8	49	21.9 [18.316, 25.484]	
Li-Jun Liu et al.	2012	8.89 ± 8.02	121	8.89 [7.461, 10.319].	
Zuo, W.	2012	21.67 ± 9.45	51	21.67 [19.0764, 24.2636]	
Lee, Y.C.	2013	16.5 ± 10.6	18	16.5 [11.603, 21.397]	Single study different group but same condition
Lee, Y.C.	2013	20.5 ± 13.5	20	20.5 [14.583, 26.417]	
Karbach, J. et al.	2014	9.42 ± 11.4	73	9.42 [6.8049, 12.0351]	
Motallebnezhad et al., 2014	2014	20.14 ± 10.92	35	20.14 [16.5223, 23.7577]	
Kunz et al.	2015	19.4 ± 10.7	10	19.4 [12.768, 26.032]	
Lopez-Jornet, P.	2016	12.88 ± 3.5	26	12.88 [11.5347, 14.2253]	
Riaz H.M.A.	2017	3.27 ± 1.17	18	3.27 [2.7295, 3.8105]	Single study different group but same condition
Riaz H.M.A.	2017	3.50 ± 1.42	18	3.5 [2.844, 4.156]	
Vilar-Villanueva, M.	2019	28.01 ± 10.7	48	28.1 [25.251, 30.949].	
Daume Linda,	2020	15.55 ± 10.6	62	15.55 [13.0434, 18.0566].	Single study different group but same condition
Daume Linda,	2020	11.06 ± 10.88	50	11.06 [8.0443, 14.0757]	
Wiriyakijja et al-IDJ	2020	15.11 ± 8.783	300	15.11 [14.1165, 16.1035]	
Partatescu et al.	2020	13.77 ± 10.83	80	13.77 [11.3968, 16.1432]	

## Data Availability

The quantitative and descriptive data supporting this Systematic Review or Meta-Analysis are from previously reported studies and datasets, which have been cited.

## Supplementary Materials

The following are available online at https://www.mdpi.com/article/10.3390/clinpract11020040/s1, Figure S1: Correlation of mean baseline OHIP score and female proportion, Figure S2: Correlation of mean baseline OHIP score and mean age, Figure S3: Correlation of publication year and baseline mean OHIP-14 score, Figure S4: Correlation of sample size and baseline mean OHIP-14 score, Table S1: PRISMA checklist, Table S2: Search strategy and search hit, Table S3: Methodological quality assessment of randomized controlled trials (RCTs) using Cochrane RoB 2 tool, Table S4: Methodological quality assessment of non-randomized controlled trials (RCTs) using MINORS (Methodological Index for Non-Randomized Studies) guidelines. All studies were judged as low-quality studies, Table S5: NIH Quality Assessment Tool for Observational Cohort and Cross-Sectional Studies.
