# Supplementary material for: Impact of Oral Lichen Planus on Oral Health-Related Quality of Life: A Systematic Review and Meta-Analysis
Source: Clin Pract. 2021 May 7;11(2):272–86. doi: 10.3390/clinpract11020040 (PMC8161446; doi:10.3390/clinpract11020040)
Supplement: Supplementary file 1 [file clinpract-11-00040-s001.zip › clinpract-1187044-supplementary.pdf]

clinics and practice

MDPI

Supplementary file

Table S1. PRISMA Checklist.

**TITLE**			
Title	1	Identify the report as a systematic review, meta-analysis, or both.	1
**ABSTRACT**			
Structured summary	2	Provide a structured summary including, as applicable: background; objectives; data sources; study eligibility criteria, participants, and interventions; study appraisal and synthesis methods; results; limitations; conclusions and implications of key findings; systematic review registration number.	1
**INTRODUCTION**			
Rationale	3	Describe the rationale for the review in the context of what is already known.	2
Objectives	4	Provide an explicit statement of questions being addressed with reference to participants, interventions, comparisons, outcomes, and study design (PICOS).	2
**METHODS**			
Protocol and registration	5	Indicate if a review protocol exists, if and where it can be accessed (e.g., Web address), and, if available, provide registration information including registration number.	NA
Eligibility criteria	6	Specify study characteristics (e.g., PICOS, length of follow-up) and report characteristics (e.g., years considered, language, publication status) used as criteria for eligibility, giving rationale.	4
Information sources	7	Describe all information sources (e.g., databases with dates of coverage, contact with study authors to identify additional studies) in the search and date last searched.	4
Search	8	Present full electronic search strategy for at least one database, including any limits used, such that it could be repeated.	5
Study selection	9	State the process for selecting studies (i.e., screening, eligibility, included in systematic review, and, if applicable, included in the meta-analysis).	5
Data collection process	10	Describe method of data extraction from reports (e.g., piloted forms, independently, in duplicate) and any processes for obtaining and confirming data from investigators.	5
Data items	11	List and define all variables for which data were sought (e.g., PICOS, funding sources) and any assumptions and simplifications made.	5
Risk of bias in individual studies	12	Describe methods used for assessing risk of bias of individual studies (including specification of whether this was done at the study or outcome level), and how this information is to be used in any data synthesis.	6
Summary measures	13	State the principal summary measures (e.g., risk ratio, difference in means).	6
Synthesis of results	14	Describe the methods of handling data and combining results of studies, if done, including measures of consistency (e.g., I^2) for each meta-analysis.	6
Risk of bias across studies	15	Specify any assessment of risk of bias that may affect the cumulative evidence (e.g., publication bias, selective reporting within studies).	5
Additional analyses	16	Describe methods of additional analyses (e.g., sensitivity or subgroup analyses, meta-regression), if done, indicating which were pre-specified.	5
**RESULTS**			
Study selection	17	Give numbers of studies screened, assessed for eligibility, and included in the review, with reasons for exclusions at each stage, ideally with a flow diagram.	6,7
Study characteristics	18	For each study, present characteristics for which data were extracted (e.g., study size, PICOS, follow-up period) and provide the citations.	6,7

Risk of bias within studies	19	Present data on risk of bias of each study and, if available, any outcome level assessment (see item 12).	8
Results of individual studies	20	For all outcomes considered (benefits or harms), present, for each study: (a) simple summary data for each intervention group (b) effect estimates and confidence intervals, ideally with a forest plot.	8
Synthesis of results	21	Present results of each meta-analysis done, including confidence intervals and measures of consistency.	6,7
Risk of bias across studies	22	Present results of any assessment of risk of bias across studies (see Item 15).	8
Additional analysis	23	Give results of additional analyses, if done (e.g., sensitivity or subgroup analyses, meta-regression [see Item 16]).	8
**DISCUSSION**			
Summary of evidence	24	Summarize the main findings including the strength of evidence for each main outcome; consider their relevance to key groups (e.g., healthcare providers, users, and policy makers).	9,10,11
Limitations	25	Discuss limitations at study and outcome level (e.g., risk of bias), and at review-level (e.g., incomplete retrieval of identified research, reporting bias).	11
Conclusions	26	Provide a general interpretation of the results in the context of other evidence, and implications for future research.	11
**FUNDING**			
Funding	27	Describe sources of funding for the systematic review and other support (e.g., supply of data); role of funders for the systematic review.	NIL

Table S2. Search strategy and search hit.

Pubmed-	(Oral lichen planus OR "Oral lichen planus" OR lichen planus OR OLP) AND (quality of life OR QoL OR "QoL" OR "oral health–related quality of life" OR OHRQoL OR OHIP-14 OR Oral Health Impact Profile)	**202**
Scilit-	ALL field ((oral lichen planus) AND (QHRQoL)) OR ((lichen planus) AND (oral health-related quality of life)) OR ((oral lichen planus) AND (OHIP-14)) OR ((oral lichen planus) AND (oral impact on daily activities))) -	47
EMBASE	(Oral lichen planus OR "Oral lichen planus" OR lichen planus OR OLP) AND (quality of life OR QoL OR "QoL" OR "oral health–related quality of life" OR OHRQoL OR OHIP-14 OR Oral Health Impact Profile)	25
Scopus	(Oral lichen planus OR "Oral lichen planus" OR lichen planus OR OLP) AND (quality of life OR QoL OR "QoL" OR "oral health–related quality of life" OR OHRQoL OR OHIP-14 OR Oral Health Impact Profile)	174
Web of Sciences	(Oral lichen planus OR "Oral lichen planus" OR lichen planus OR OLP) AND (quality of life OR QoL OR "QoL" OR "oral health–related quality of life" OR OHRQoL OR OHIP-14 OR Oral Health Impact Profile)	85
Google Scholar	(Oral lichen planus OR "Oral lichen planus" OR lichen planus OR OLP) AND (quality of life OR QoL OR "QoL" OR "oral health–related quality of life" OR OHRQoL OR OHIP-14 OR Oral Health Impact Profile)	260
Clinical Trial Registry	Oral lichen planus, OHIP-14	26

Table S3. Methodological quality assessment of of randomized controlled trials (RCTs) using Cochrane RoB 2 tool.

Study	Domain					
	**Randomization process**	**Deviations from the intended interventions**	**Missing outcome data**	Measurement of the outcome	Selection of the reported result	Overall Bias
Hegarty et al 2002	Low	Low	High	Low	Low	High
Chan L Y et al 2013	Some concerns	Low	Low	Low	Low	Some concerns
Lopez Jornet et al 2016	Low	Low	Low	Low	Low	Low
Riaz H M A et al 2017	Some concerns	Low	Low	Low	Low	Some concerns

1 = Low; 1= High; 2 = Some Concerns

Table S4. Methodological quality assessment of non-randomized controlled trials (RCTs) using MINORS (Methodological Index for Non-Randomized Studies) guidelines. All studies were judged as low quality.

**Methodological items for non-randomized studies (MINORS)**	**Score†**		
	**Kaliakatsou et al 2002**	**Macgrath et al 2003**	**Kunz et al 2014**
1. **A clearly stated aim:** the question addressed should be precise and relevant in the light of available literature	2	2	2
2. **Inclusion of consecutive patients**: all patients potentially fit for inclusion (satisfying the criteria for inclusion) have been included in the study during the study period (no exclusion or details about the reasons for exclusion)	0	2	2
3. **Prospective collection of data**: data were collected according to a protocol established before the beginning of the study	2	2	2
4. **Endpoints appropriate to the aim of the study**: unambiguous explanation of the criteria used to evaluate the main outcome which should be in accordance with the question addressed by the study. Also, the endpoints should be assessed on an intention-to-treat basis.	2	2	2
5. **Unbiased assessment of the study endpoint**: blind evaluation of objective endpoints and double-blind evaluation of subjective endpoints. Otherwise the reasons for not blinding should be stated	0	2	0
6. **Follow-up period appropriate to the aim of the study**: the follow-up should be sufficiently long to allow the assessment of the main endpoint and possible adverse events	2	2	2
7. **Loss to follow up less than 5%**: all patients should be included in the follow up. Otherwise, the proportion lost to follow up should not exceed the proportion experiencing the major endpoint	2	2	0
8. **Prospective calculation of the study size**: information of the size of detectable difference of interest with a calculation of 95% confidence interval, according to the expected incidence of the outcome event, and information about the level for statistical significance and estimates of power when comparing the outcomes	0	0	0
Additional criteria in the case of comparative study			
9. **An adequate control group**: having a gold standard diagnostic test or therapeutic intervention recognized as the optimal intervention according to the available published data	NA	0	0
10. **Contemporary groups**: control and studied group should be managed during the same time period (no historical comparison)	NA	0	0
11. **Baseline equivalence of groups**: the groups should be similar regarding	NA	0	0

the criteria other than the studied endpoints. Absence of confounding factors that could bias the interpretation of the results			
12. **Adequate statistical analyses**: whether the statistics were in accordance with the type of study with calculation of confidence intervals or relative risk	NA	0	0
Total score	10	14	10

† The items are scored 0 (not reported), 1 (reported but inadequate) or 2 (reported and adequate). The global ideal score being 16 for non-comparative studies and 24 for comparative studies.

Table S5. NIH tool- Quality Assessment Tool for Observational Cohort and Cross-Sectional Studies (https://www.nhlbi.nih.gov/health-topics/study-quality-assessment-tools).

Criteria	Llewellyn S et al	Tabolli S et al	Li-Jun Liu et al	Karbach J et al	Vilar-Villanueva M et al	Daume L et al	Parlatescu et al	Zuo W et al	Wiriyakijja et al	Motallebnezhad et al
1. Was the research question or objective in this paper clearly stated?	Y	Y	Y	Y	Y	Y	Y	Y	Y	Y
2. Was the study population clearly specified and defined?	Y	Y	Y	Y	Y	Y	Y	Y	Y	Y
3. Was the participation rate of eligible persons at least 50%?	Y	Y	Y	Y	Y	Y	Y	CD	Y	Y
4. Were all the subjects selected or recruited from the same or similar populations (including the same time period)? Were inclusion and exclusion criteria for being in the study prespecified and applied uniformly to all participants?	Y	Y	Y	Y	Y	Y	Y	Y	Y	Y
5. Was a sample size justification, power description, or variance and effect estimates provided?	NR	NR	NR	NR	NR	NR	NR	NR	NR	NR
6. For the analyses in this paper, were the exposure(s) of interest measured prior to the outcome(s) being measured?	Y	Y	Y	Y	Y	Y	Y	Y	Y	Y
7. Was the timeframe sufficient so that one could reasonably expect to see an association between exposure and outcome if it existed?	Y	Y	Y	Y	Y	Y	Y	Y	Y	Y
8. For exposures that can vary in amount or level, did the study examine different levels of the exposure as related to the outcome (e.g., categories of exposure, or exposure measured as continuous variable)?	Y	Y	Y	Y	Y	Y	Y	Y	Y	Y
9. Were the exposure measures (independent variables) clearly defined, valid, reliable, and implemented consistently across all study participants?	Y	Y	Y	Y	Y	Y	Y	Y	Y	Y
10. Was the exposure(s) assessed more than once over time?	N	N	N	N	N	N	N	N	N	N
11. Were the outcome measures (dependent variables) clearly defined, valid, reliable, and implemented consistently across all study participants?	Y	Y	Y	Y	Y	Y	Y	Y	Y	Y
12. Were the outcome assessors	NR	NR	NR	NR	NR	NR	Y	CD	NR	NR

blinded to the exposure status of participants?										
13. Was loss to follow-up after baseline 20% or less?	N	N	N	N	N	N	N	N	N	N
14. Were key potential confounding variables measured and adjusted statistically for their impact on the relationship between exposure(s) and outcome(s)?	Y	NR	NR	NR	NR	Y	Y	Y	Y	Y
**Quality Rating (Good, Fair, or Poor)**	Good	Good	Good	Good	Good	Good	Good	Fair	Good	Good

CD, cannot determine; NA, not applicable; NR, not reported; Y, Yes; N, No.

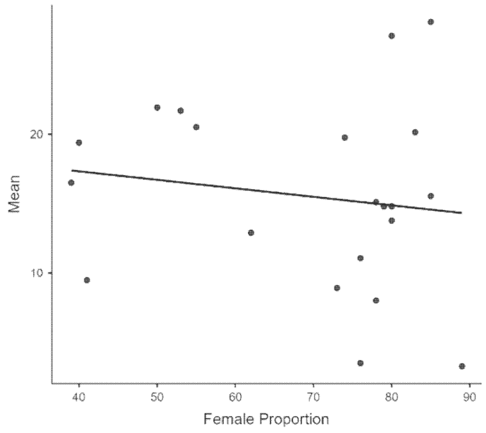

Figure S1. Correlation of Mean baseline OHIP Score and female proportion.

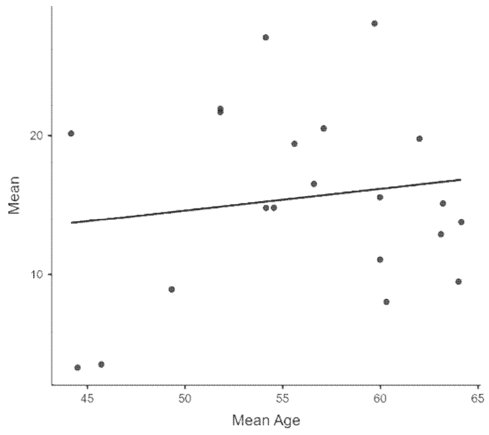

Figure S2. Correlation of mean baseline OHIP score and mean Age.

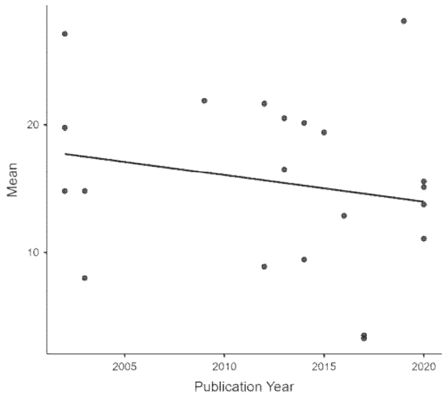

Figure S3. Correlation of Publication year and baseline mean OHIP-14 Score.

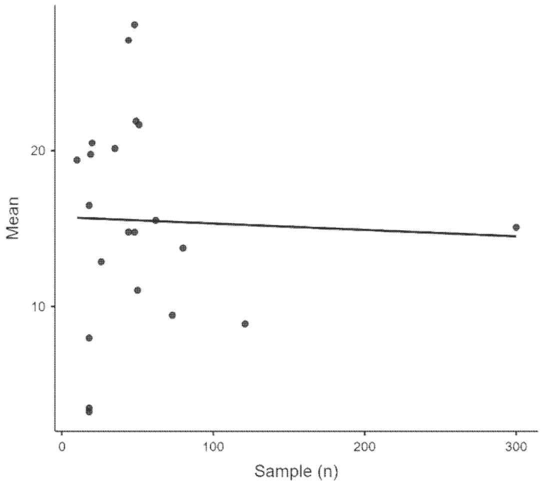

Figure S4. Correlation of sample size and baseline mean OHIP-14 score.
